# Pharmacological modulation of MRAP2 protein on murine melatonin receptor signaling

**DOI:** 10.3389/fendo.2025.1593345

**Published:** 2025-05-29

**Authors:** Wenqi Song, Yanchuan Li, Hongtao Xu, Yaqun Zhang, Liu Liu, Yihao Li, Xinran Wang, Yueming Du, Yining Chen, Jianjun Lyu, Lingjing Jin, Chao Zhang, Yuchen Xiao

**Affiliations:** ^1^ Fundamental Research Center, Shanghai Yangzhi Rehabilitation Hospital (Shanghai Sunshine Rehabilitation Center), School of Life Sciences and Technology, Tongji University, Shanghai, China; ^2^ Hubei Topgene Research Institute of Hubei Topgene Biotechnology Group Co., Ltd., Wuhan, China; ^3^ Department of Orthopedics and Precision Research Center for Refractory Diseases, Shanghai General Hospital, Shanghai Jiao Tong University School of Medicine, Shanghai, China; ^4^ Department of Neuroscience, School of Basic Medical Science, Soochow University, Suzhou, Jiangsu, China; ^5^ Shanghai Yuhui Pharmaceutical Technology (Group) Co., Ltd., Shanghai, China; ^6^ The Hong Kong University of Science and Technology (HKUST), Hong Kong, Hong Kong SAR, China; ^7^ Beijing No.8 High School, Beijing, China; ^8^ The Experimental High School Attached Beijing Normal University (EHS-BNU), Beijing, China; ^9^ Fundamental Research Center, Shanghai Yangzhi Rehabilitation Hospital (Shanghai Sunshine Rehabilitation Center), School of Medicine, Tongji University, Shanghai, China

**Keywords:** MTNR1A, MTNR1B, MRAP2, melatonin, metabolism

## Abstract

**Introduction:**

MTNR1A and MTNR1B, crucial for regulating circadian rhythms and various physiological processes, have well- established biological significance. The role of MRAP2, a single transmembrane accessory protein, in modulating the pharmacological activity of melatonin receptors remains unclear.

**Methods:**

We first examined the evolutionary profiles of melatonin receptors and MRAP2 by protein sequence alignment and synteny analysis. Bulk RNA-seq was then employed to analyze the expression distribution of these genes. Next, we performed co-immunoprecipitation and Bimolecular Fluorescence Complementation (BiFC) assays to investigate the interaction of MRAP2 with melatonin receptors. We also recruited the GloSensor luminescence assay to assess the impact of MRAP2 on the Gi signaling pathway of melatonin receptors, and conducted fixed-cell ELISA to evaluate MRAP2’s effect on melatonin receptor membrane trafficking.

**Results:**

Our results revealed that MTNR1A was most conserved in terms of evolution, while all of these genes showed adaptive changes in amphibians and zebrafish likely due to aquatic environment. MRAP2 was found to inhibit the constitutive activity of melatonin receptors and enhance their maximal agonist potency. Additionally, MRAP2 suppressed the membrane trafficking of MTNR1A, but promoted the surface trafficking of MTNR1B.

**Discussion:**

These findings highlighted the complex regulatory role of MRAP2, and shed light on its diverse functions in GPCR biology and its potential implications in regulating physiological processes governed by melatonin signaling.

## Introduction

G protein-coupled receptors (GPCRs) function as essential players in diverse physiological processes, including neurotransmission, hormone regulation, and metabolic homeostasis as ‘molecular switches’ for cellular signal transduction (1). Emerging paradigms in GPCR biology reveal that receptor functionality is frequently modulated through interactions with auxiliary proteins. The melanocortin receptor accessory protein (MRAP) family exemplifies this regulatory mechanism, where MRAP1 was first recognized as an essential chaperone facilitating MC2R transport and adrenocorticotropic hormone (ACTH) responsiveness (2–4). Subsequent studies uncovered broader regulatory roles for MRAP2, demonstrating its capacity to bidirectionally modulate melanocortin receptor subtypes (4). MRAP2 has been reported to regulate the function of central melanocortin receptors MC3R and MC4R signaling cascades in a flexible manner across various vertebrate species, including human (4, 5), mouse (6), clawed frog (7–9), axolotl (10), and Nile tilapia (11). Notably, in zebrafish, MRAP2a inhibiting its function to promote early growth, while MRAP2b enhances α-MSH sensitivity after feeding to support regulated growth (12). MRAP2a and MRAP2b can also regulate MC4R activity as heterodimers (13). For other melanocortin receptors, MRAP2 suppresses MC5R signaling by inhibiting its cell surface expression and reducing receptor efficacy to α-MSH and SHU9119, potentially through altered receptor trafficking and oligomerization dynamics, rather than clathrin-mediated internalization (14). Both MRAP2a and MRAP2b can interact with MC5Ra and MC5Rb receptors and regulate their cell surface expression in zebrafish (15). Recent genomic analyses have revealed evolutionary complexity in MRAP2 biology. In humans and canines, alternative splicing generates MRAP2 isoforms with distinct C-terminal domains that differentially regulate receptor pharmacology (4, 14). While mouse *MRAP2* splice variants remain uncharacterized, phylogenetic conservation of splice sites suggests potential functional diversification across species. The expanding understanding of MRAP-mediated GPCR regulation provides a critical context for investigating novel receptor-modulator interactions. Beyond melanocortin receptors, MRAP2 modulates diverse GPCR families: it amplifies ghrelin receptor (GHSR1a) sensitivity to acyl-ghrelin while suppressing orexin receptor (OX1R) constitutive activity through distinct structural domains (16–18). MRAP2 can also inhibit melanin-concentrating hormone receptor (MCHR1) signaling *in vitro (* 19). Such bidirectional regulation extends to metabolic GPCRs like prokineticin receptors, where MRAP2 inhibits PKR1 surface expression but enhances PKR2 ligand responsiveness (2, 16, 18, 20). These precedents establish MRAP2 as a master regulator of GPCR signaling landscapes, making its interaction with melatonin receptors a logical extension of its functional repertoire. Building on MRAP2’s established capacity to influence GPCR trafficking, dimerization dynamics, and ligand responsiveness, we hypothesize that it may regulate melatonin receptor signaling similarly. However, the mechanistic nature of MRAP2 interactions with melatonin receptors (MTNR1A/MTNR1B) remains undefined. Clarifying these interactions could enhance insights into the broader role of MRAP2 in GPCR biology and its potential implications for melatonin-associated physiological function.

Melatonin is predominantly produced in the pineal gland and is additionally present in multiple diverse tissues, including bone marrow, cerebellum, skin, lymphocytes, mammary gland, and gastrointestinal tract. It follows a circadian rhythm, regulating hormonal levels accordingly (21–25). The suprachiasmatic nucleus (SCN) in the hypothalamus governs melatonin synthesis by integrating optical stimuli from retina with changes in natural light (26, 27). Interspecies variations exist in regulating melatonin synthesis, evidenced by contrasting gene expression patterns in zebrafish pineal gland cells during the day and in mammals (28). Melatonin has been demonstrated to suppress cAMP formation and regulate PKA function by Gi protein (29). The central nervous system exerts antioxidant effects, protecting neurons from oxidative stress and regulates blood pressure and cardiovascular autonomic function (30–32). Melatonin, with its antioxidant effects, inhibit the development of various tumors (33–36). Melatonin receptors MTNR1A and MTNR1B have attracted much attention due to their unique circadian regulatory capabilities and pivotal metabolic roles, which are encoded by distinct genes located on chromosomes 4q35.1 and 11q21-q22, respectively (37). As products of multiple gene duplication and rearrangement events in early vertebrate evolution, they share high sequence homology but exhibit significant evolutionary divergence in functional specialization and tissue distribution (38). Phylogenetic analyses reveal that their conserved transmembrane domains (e.g., Y5.58, H6.54) maintain key melatonin-binding sites, while sequence variations in intracellular loops may drive signaling pathway specificity (39).

MTNR1A, functioning as the ‘central commander’ of circadian rhythm, is predominantly localized in the SCN of the hypothalamus, pituitary, hippocampus, and pancreatic α-cells (40). Its functional hierarchy in the SCN manifests through circadian dynamics, seasonal adaptation, and gene regulatory networks. Firstly, MTNR1A mRNA in SCN neurons is significantly upregulated nocturnally, synchronizing with melatonin secretion peaks to drive the Gq-PKC-PLC signaling cascade, thereby suppressing neuronal electrical activity, reducing core body temperature, and promoting sleep initiation (40). Under short-day photoperiods, MTNR1A expression in the ovine hypothalamic pre-mammillary nucleus (PMH) increases 2-3-fold, enhancing melatonin sensitivity to regulate seasonal reproductive transitions (41). MTNR1A activation represses clock genes (e.g., PER1/CRY1), whereas the CLOCK/BMAL1 complex reciprocally modulates its transcription, forming a bidirectional feedback loop. Notably, MTNR1A knockout models exhibit complete abolition of SCN neuronal firing rhythmicity, underscoring its indispensable role as the biological clock’s pacemaker (41).

In contrast to MTNR1A’s central dominance, MTNR1B serves as a ‘precision regulator’ of metabolic homeostasis, primarily expressed in peripheral metabolic organs: retina, pancreatic β-cells, liver, and adipose tissue, associated with glucose metabolism (42). Despite having only 20% of MTNR1A’s mRNA abundance, MTNR1B exerts potent suppression of glucose-stimulated insulin secretion (GSIS) via Gi/o signaling (43). The rs10830963(G) risk allele, identified in genome-wide association studies (GWAS), upregulating receptor expression, conferring β-cell hypersensitivity to melatonin (OR=1.15, p=6×10^-^¹³), that allele’s higher prevalence in East Asian populations (43% vs. 28% in Europeans) correlates with a 1.4-fold rise in fasting hyperglycemia (42). Nocturnal melatonin inhibits lipolytic enzyme activity through MTNR1B, synergizing with insulin’s nocturnal trough to optimize energy storage. Clinical evidence indicates that delaying dinner beyond 20:00 exacerbates this pathway, elevating impaired glucose tolerance (IGT) incidence by 37% (42, 43). Although both receptors engage Gi/o signaling, tissue-specific pathway bifurcation (e.g., MTNR1A activating Gq-PLCβ3 vs. MTNR1B coupling to Gβγ-PI3Kγ) enables functional complementarity. MTNR1A maintains nocturnal hepatic glucose output by curbing α-cell glucagon secretion; MTNR1B prevents nocturnal hypoglycemia via β-cell insulin suppression (42). Disruption of the equilibrium triggers cascading pathologies, including sleep disorders, type 2 diabetes, and neurodegeneration (44–46). For instance, Alzheimer’s disease patients exhibit a 40% reduction in cerebrospinal MTNR1A levels, strongly correlating with circadian disruption severity (45–47).

Extensive studies have focused on melatonin receptor pharmacology and intracellular signaling, but little is known on their regulatory mechanisms involving accessory proteins. Emerging evidence suggests that GPCRs do not function solely as monomeric entities but can form heterodimers or interact with auxiliary proteins that modulate their localization, ligand sensitivity, and signaling efficiency. However, the regulatory landscape of melatonin receptors remains largely unexplored, warranting further investigation into their potential interactions with GPCR-modulating proteins.

Despite accumulating evidence of MRAP2’s pleiotropic GPCR regulatory effects, critical knowledge gaps persist regarding its direct physical interaction with MTNR1A/MTNR1B and the functional consequences of such interactions. We implemented an integrated experimental strategy combining biochemical, cellular, and pharmacological approaches to investigate these mechanisms. Co-immunoprecipitation (Co-IP) and confocal microscopy assessed physical interaction and subcellular co-localization between MRAP2 and melatonin receptors. Cell surface ELISA assays quantified MRAP2’s effects on receptor trafficking and plasma membrane localization. Complementary GloSensor cAMP functional assays evaluated MRAP2-mediated modulation of melatonin receptor signaling potency and efficacy. This multidimensional analysis aims to establish whether MRAP2 forms functional heterodimers with MTNR1A/MTNR1B, thereby governing their spatial distribution and signaling properties, which provide transformative insights into MRAP2’s functional plasticity in GPCR regulation and its potential roles in circadian/metabolic integration.

## Materials and methods

### Reagents, plasmids and antibodies

Mouse *Mtnr1a*, *Mtnr1b*, and *Mrap2* fragments were cloned into pcDNA3.1(+) plasmid and verified via DNA sequencing. The pGloSensor-22F cAMP plasmid and GloSensor cAMP reagent were procured from Promega (Madison, WI, USA), while melatonin was synthesized by MedChemExpress (Monmouth Junction, NJ, USA). Hydrochloric acid and sulfuric acid were sourced from Sinopharm Chemical Reagent Co., Ltd (Beijing, China). Non-fat milk powder, paraformaldehyde (4%), bovine serum albumin, and phosphate-buffered saline (PBS) were purchased from Sangon Biotech (Shanghai, China). Tetramethylbenzidine (TMB) chromogen solution and enhanced chemiluminescence reagent were obtained from Beyotime Biotechnology (Shanghai, China). Antifade reagent with DAPI and Rabbit anti-HA antibody was obtained from Cell Signaling Technology (Danvers, MA, USA). The antibodies used in this study: Mouse anti-Flag (ABclonal Biotech Co., Ltd, Wuhan, China), Rabbit anti-Flag (Proteintech, Wuhan, China), Goat anti-Mouse Alexa-Fluor 594 (Abcam, Cambridge, UK), and Goat anti-Rabbit IgG (HRP-conjugated) (ABclonal Biotech Co., Ltd, Wuhan, China).

### Multiple protein sequence alignment, phylogenetic, and synteny analysis

MTNR1A, MTNR1B, and MRAP2 protein sequences from humans, mice, painted turtles, chicken, clawed frogs, zebrafish, and elephant sharks were obtained from the NCBI (http://www.ncbi.nlm.nih.gov/) or ENSEMBL databases (http://www.ensembl.org/) in FASTA format. Sequences were aligned using ClustalW in MEGA11, and a phylogenetic tree was constructed using the Neighbor-Joining method with the JTT protein model and a bootstrap test of 1000 replications (48). For synteny analysis, adjacent genes of the target genes were manually identified and annotated by searching the NCBI and ENSEMBL databases. The specific sequence information is provided in **Supplementary Table 1**.

### Analysis alternative splicing analysis of mouse MRAP2

To explore alternative splicing events in mouse MRAP2, we accessed the annotated transcript variants from the Ensembl genome database (GRCm39 assembly). We obtained sequences for three isoforms: Mrap2-201 (ENSMUST00000049457.14), Mrap2-202 (ENSMUST00000113149.8), and Mrap2-203 (ENSMUST00000179313.3). We systematically aligned these sequences using Clustal Omega to elucidate their exon-intron structures, coding sequences (CDS), and untranslated regions (UTR). We performed a comparative nucleotide sequence analysis to verify exon structures and splicing junctions and visualized the results with SnapGene software. This analysis enabled us to identify and annotate conserved domains, including transmembrane domains, glycosylation sites, and functional motifs (YEYY and LKAHKYS) across all transcript variants. Exon usage and exon-intron boundaries were accurately annotated utilizing Ensembl exon IDs (ENSMUSE00000235341, ENSMUSE00000235357, ENSMUSE00000694502, and ENSMUSE00000694506). The study focused on discerning differences between isoforms in coding and non-coding regions. Visualized and annotated using the Integrative Genomics Viewer (IGV, Broad Institute; http://software.broadinstitute.org/software/igv/), transcript variations, exon-intron structure, and motif patterns were validated by comparing genomic coordinates and exon counts with NCBI RefSeq and Ensembl records to confirm accuracy.

### Bulk RNA-seq analysis

RNA-seq data (accession code: E-MTAB-6798) encompassing six mouse tissues were obtained from the ArrayExpress database (14). Gene expression quantification was generated to transcript-per-million (TPM) values. Expression levels of Mrap2 (ENSMUSG00000037940), Mtnr1a (ENSMUSG00000037942), and Mtnr1b (ENSMUSG00000040306) were extracted from normalized matrices. Visualizations were created with ggplot2 in R.

### Cell culture and transfection

Dulbecco’s Modified Eagle Medium (DMEM) medium with 10% (v/v) fetal bovine serum (FBS) and 1% (v/v) penicillin-streptomycin was used to culture the human embryonic kidney (HEK293T) cells at 37°C under 5% CO2 in a humidified atmosphere. Following the supplier’s instructions, the transfection was performed using polyethyleneimine reagent (PEI, Yeasen, Shanghai, China). An empty pcDNA3.1(+) vector was added to each transfection mixture to maintain consistent plasmid quantities.

### Co-immunoprecipitation and western blot

HEK293 cells were seeded at a density of 1×10^5^cells/mL in a 6-well plate and transfected with 2µg of the specified plasmid per well once they reached 50–60% confluence. MTNR1A and MTNR1B were integrated into the pcDNA3.1(+) plasmid with a Flag protein tag at the N-terminus, while MRAP2 was cloned into the pcDNA3.1(+) plasmid with HA protein tag at the N-terminus. Flag-MTNR1A or Flag-MTNR1B (1μg DNA/well) were co-transfected with HA-MRAP2 (1μg DNA/well) into HEK293 cells. Following a 36-hour incubation, cells were rinsed with PBS and lysed at 4°C for one hour using a western/IP lysis buffer (Beyotime Biotechnology, Shanghai, China). Centrifugation was performed to eliminate insoluble material. The resulting supernatant was evenly divided into two portions, one portion was incubated with Flag Nanoab Agarose beads (NuoYi Biotech, Tianjin, China) for 2 hours at 4°C with gentle rotation, the other portion was kept for input samples. Flag Nanoab Agarose beads captured flag-tagged proteins or protein complexes. Post-incubation, the beads underwent three washes with lysis buffer before elution. Protein samples were combined with SDS Sampling Buffer with 5% β-mercaptoethanol, then heated at 65°C for 15 minutes. Proteins were denatured, resolved on a 12% SDS-PAGE gel, and transferred onto a PVDF membrane. For immunoblotting, membranes were probed with either rabbit anti-HA or anti-Flag primary antibodies, followed by incubation with HRP-conjugated goat anti-rabbit secondary antibodies. Using enhanced chemiluminescence reagents, Protein bands were detected with an ImageQuant LAS 4000 imaging system (General Electric Company, CT, USA).

### Bimolecular fluorescence complementation assay

HEK293T cells were plated into a 12-well dish on cover glasses pre-treated with 0.01% poly-D-lysine at a concentration of 1×10^5^cells/mL. Mouse MTNR1A and MTNR1B plasmids were fused with Venus fragment 1 (VF1) at the C-terminus, while a Flag protein tag was attached to the N-terminus. Venus fragment 2 (VF2) was attached to the C terminus of the mouse MRAP2 plasmid. Flag-MTNR1A-VF1 or Flag-MTNR1B-VF1 (1μg DNA/well) were co-transfected with Flag-MRAP2-VF2 (1μg DNA/well) into HEK293 cells when the cell density reached 60%. Following a 24-hour transfection, cells were fixed with 4% paraformaldehyde for 15 minutes after removing the culture medium. After fixation, cells underwent three PBS washes. To prevent nonspecific interactions, a blocking solution containing 0.1% Triton X-100, 5% sheep serum, and 94.9% PBS was applied for permeabilization to prevent nonspecific interactions. Permeable cells with permeability were incubated with Mouse anti-Flag monoclonal antibody (1:2000) for 2 hours at room temperature, followed by three washes. Subsequently, Goat anti-Mouse Alexa-Fluor 594 (1:2000) was applied as a secondary antibody for 2 hours in the dark. Coverslips were subsequently fixed using an antifade mounting medium containing DAPI. Confocal microscopy imaging was conducted on a Nikon confocal microscope.

### cAMP luminescent assay

HEK293 cells were initially plated at a concentration of 1×10^5^cells/mL, and transfected once cellular density reached 50-60%. The plasmids encoding mouse MTNR1A or MTNR1B and MRAP2 at varying ratios(1:0, 1:1, 1:3, and 1:6) in conjunction with an empty pcDNA3.1(+) vectors were transiently co-transfected into HEK293T cells at a total DNA amount of 1µg per well. Additionally, 1µg of the pGloSensor-22F cAMP plasmid was included to monitor cAMP production. Following a 36-hour incubation, cells were detached, transferred to white 96-well plates, followed by incubation for another 4-6 hours. Subsequently, the medium was substituted with an equilibration solution comprising 88% CO_2_-independent medium, 10% fetal bovine serum, and 2% GloSensor cAMP reagent. It was then incubated at room temperature for 2 hours. Luminescence was assessed in kinetic cycle mode by a microplate reader (Thermo Fisher, MA, USA), with exposure every 30 seconds for 10–15 minutes to establish baseline luminescence values. Different melatonin concentrations were added to each well and mixed thoroughly, followed by continued luminescence measurement under the same kinetic cycle mode for an additional 20–30 minutes. Luminescence values recorded after the addition of the agonist were adjusted based on the baseline measurement.

### Fixed-cell ELISA for surface epitope detection

HEK293T cells were seeded in 24-well plates pre-coated with 0.01% poly-D-lysine at a density of 1×10^5^cells/mL. MTNR1A and MTNR1B were constructed into pcDNA3.1(+) plasmid with Flag protein tag at the N-terminus, while MRAP2 was constructed into pcDNA3.1(+) plasmid with HA protein tag at the N-terminus. Upon reaching 60% confluency, cells were co-transfected with Flag-tagged MTNR1A or MTNR1B and HA-MRAP2 at varying ratios (1:0, 1:3, and 1:6). Blocking was performed for 40 min with 5% non-fat milk in D-PBS, after which cells were probed with Rabbit anti-Flag antibody for 2 h at room temperature. After further washes, cells were incubated with HRP-conjugated Goat anti-Rabbit IgG (1:200 dilution) for 2 h at room temperature. Finally, after additional washing steps, TMB substrate was added for 15 min in the dark. The addition of 2 mol/L sulfuric acid terminated the reaction. Thermo Fisher microplate reader recorded the absorbance at 450 nm.

## Results

### Multiple protein sequence alignment, synteny, and phylogenetic analysis of MTNR1A, MTNR1B and MRAP2

**Figure 1:**
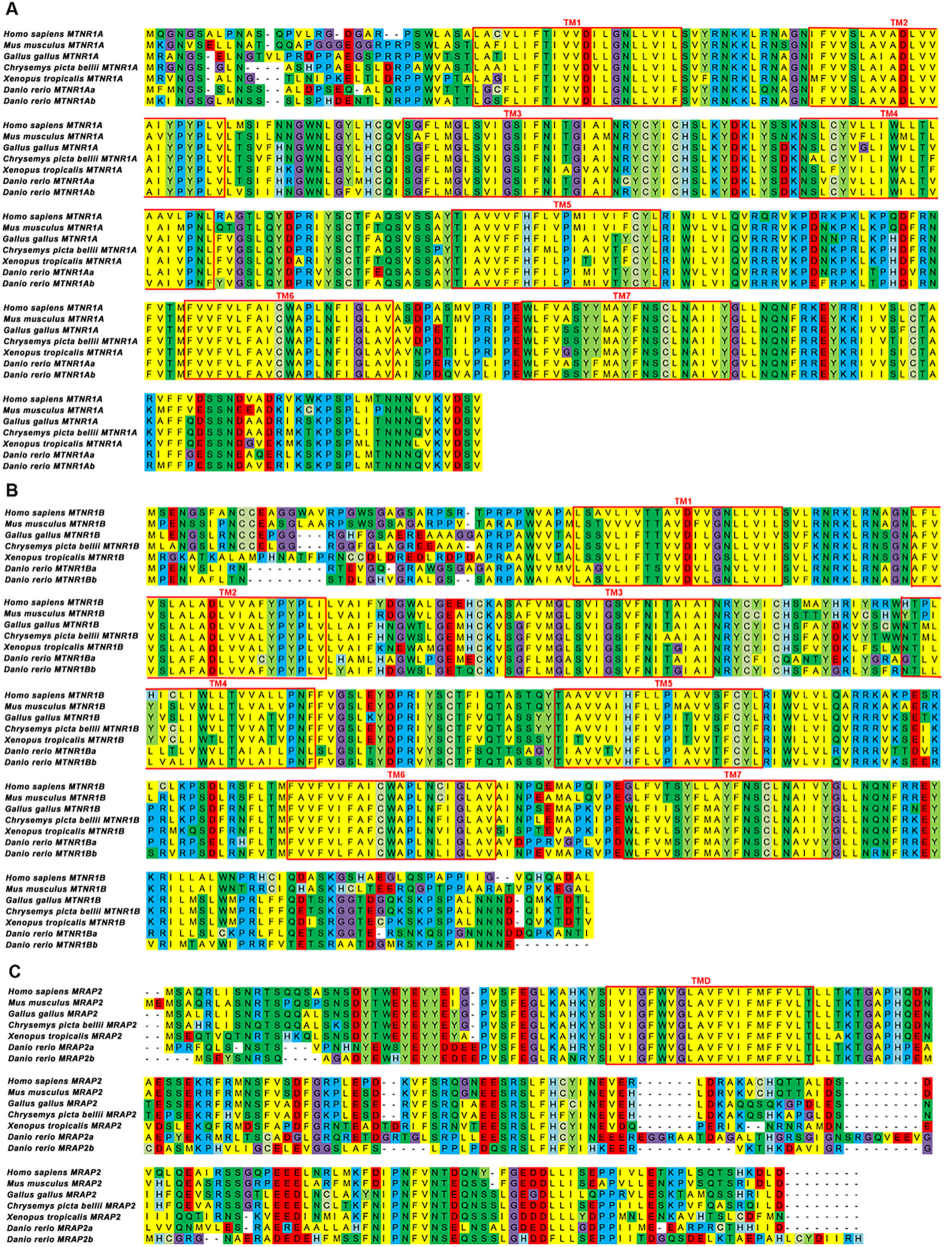
Sequence alignments of multiple MTNR1A, MTNR1B, and MRAP2 proteins. **(A)** Sequence alignment of multiple MTNR1A proteins. **(B)** Sequence alignment of multiple MTNR1B proteins. **(C)** Sequence alignment of multiple MRAP2 proteins. The transmembrane (TM) regions in the MTNR1A and MTNR1B are represented by red box and are numbered 1–7. The red box marks the transmembrane domains (TMD) in the MRAP2.

The protein sequences of mouse MTNR1A, MTNR1B, and MRAP2 were aligned across representative vertebrate classes, including primate (human), reptile (painted turtle), teleost (zebrafish), avian (chicken), amphibia(African clawed frog), and rodent (mouse), as shown in **Figures 1A–C**. Notably, all three genes had paralogs in zebrafish. Mouse MTNR1A and its orthologs exhibited an average sequence identity of 78%, with the highest identity observed in human (84%) and the lowest in zebrafish MTNR1AA (73%) (**Figure 1A**). MTNR1B shared the lowest average identity (63%) with its orthologs, with the highest identity to human (79%) and the lowest to zebrafish MTNR1BA (56%) (**Figure 1B**). The average sequence identity of MRAP2 and its orthologs was comparatively low (64%), showing the highest similarity to the human counterpart (87%) and the weakest to zebrafish MRAP2B (43%) (**Figure 1C**). In summary, MTNR1A showed moderate conservation across species, with its non-conserved region primarily situated at the N-terminus. MTNR1B and MRAP2 exhibited some sequence variability, showing non-conserved segments at both termini, while their similarity was primarily concentrated in the transmembrane regions.

**Figure 2:**
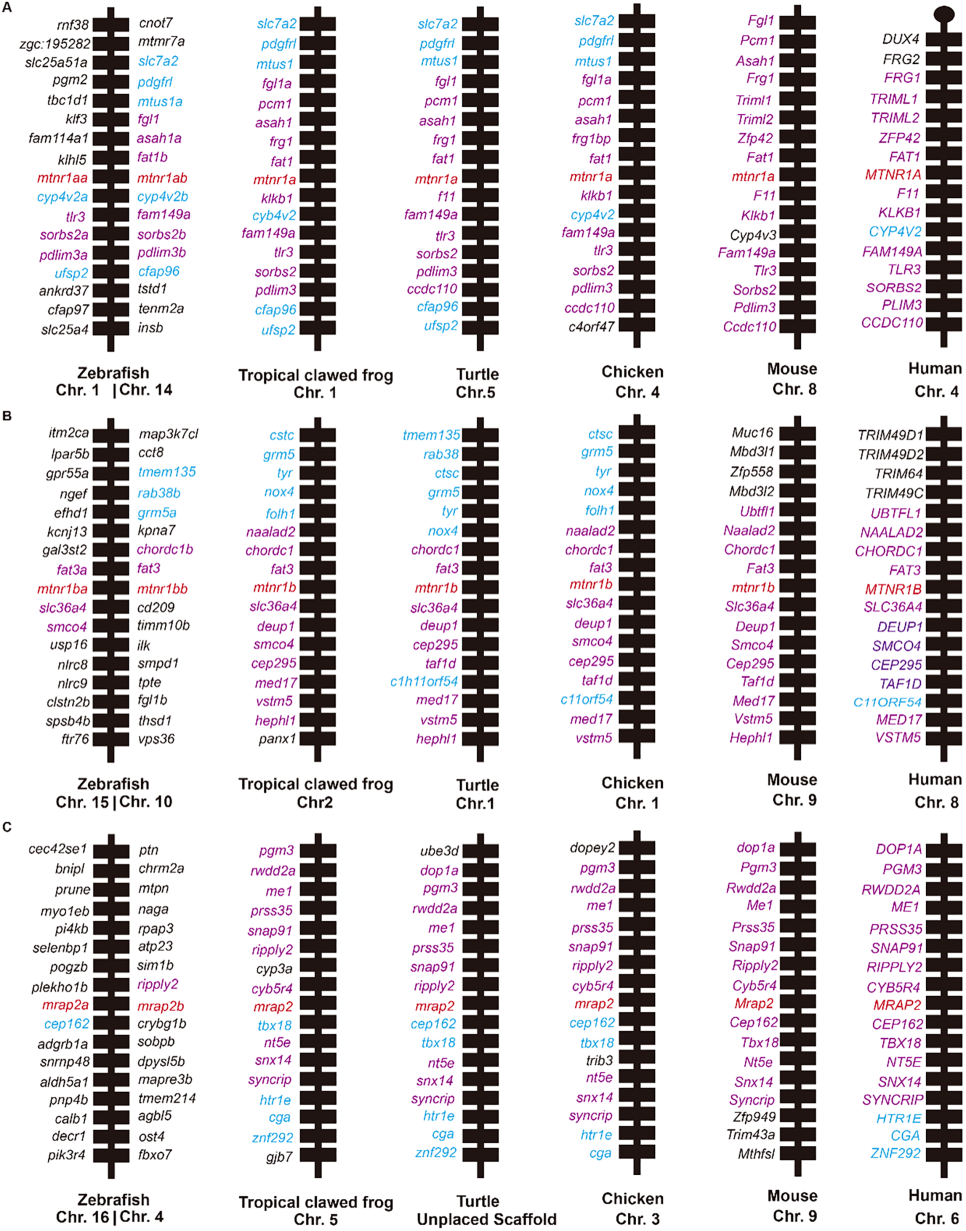
Synteny analysis of *Mtnr1a*, *Mtnr1b*, and *Mrap2*. Chromosomal location and adjacent genes of **(A)** *Mtnr1a*, **(B)** *Mtnr1b*, and **(C)** *Mrap2* in following species: mammal (Human, Mouse), avian (Chicken), reptile (Turtle), amphibia (Tropical clawed frog), and teleost (Zebrafish). Genes showing conserved synteny with mouse are shown in purple. Genes showing conserved synteny among at least two species but not with mouse are shown in blue.

Synteny analysis provided additional insights into the homology of *Mtnr1a*, *Mtnr1b*, and *Mrap2* across the aforementioned species (**Figure 2**). All flanking genes of mouse *Mtnr1a* except *Cyp4v3* were conserved across other species (highlighted in purple, **Figure 2A**). The syntenic block of *Mtnr1a* in mouse was homologous in all species except chromosome 1 in zebrafish. In addition, the degree of conservation decreases in more distant species related to mice. For mouse *Mtnr1b*, the two upstream flanking genes *Chordc1* and *Fat3* were conserved in all species (highlighted in purple, **Figure 2B**), while other upstream flanking genes like *Muc16*, *Mbd3l1*, *Zfp558* and *Mbd3l2* were primarily conserved across all species except zebrafish. Similarly, the syntenic regions surrounding mouse *Mrap2* remained broadly except in zebrafish (**Figure 2C**). Nevertheless, the downstream flanking genes *Zfp949*, *Trim43a*, and *Mthfsl* were unique to the mouse. Overall, the adjacent genes of *Mtnr1a*, *Mtnr1b*, and *Mrap2* were conserved in clawed frogs, turtles, chickens, mice, and humans. The specific neighboring genes and two distinct copies in zebrafish suggested a unique evolutionary pattern in teleosts (10).

**Figure 3:**
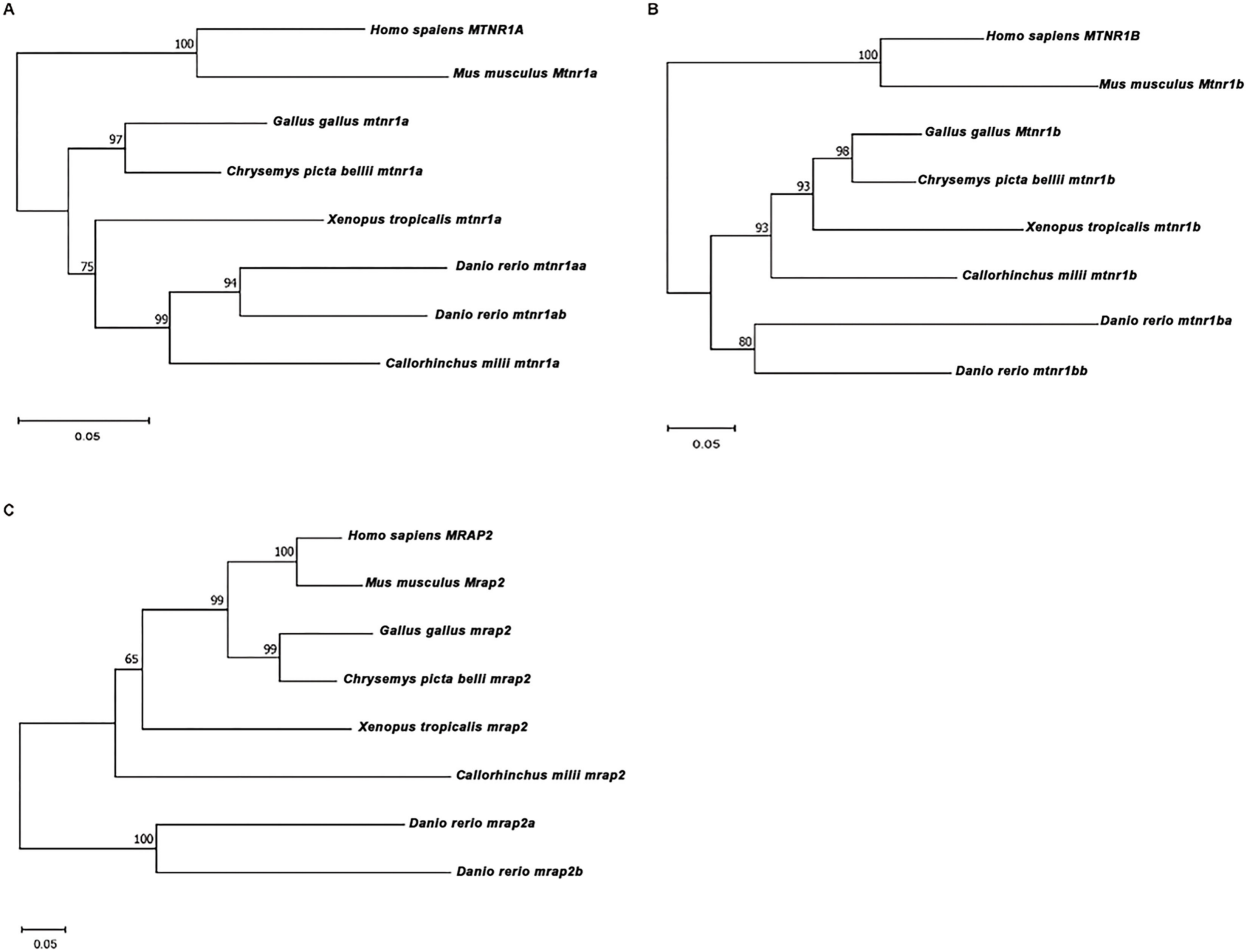
Phylogenetic analysis of MTNR1A, MTNR1B, and MRAP2. Phylogenetic trees of **(A)** MTNR1A, **(B)** MTNR1B, and **(C)** MRAP2. The Neighbor-Joining (NJ) phylogenetic tree was inferred based on multiple alignments of protein sequences using MEGA 11 with the JTT protein model and 1,000 rapid bootstrap replicates.

To further elucidate understanding of the evolutionary connections among these melatonin receptors, the NJ phylogenetic trees were constructed using protein sequences from species mentioned above, along with cartilaginous fish (elephant shark) (**Figure 3**). MTNR1A, MTNR1B, and MRAP2 were most closely related to human with a high bootstrap value of 99-100 — the evolutionary clades of most species aligned well with their phylogenetic relationships. Notably, the MTNR1B and MRAP2 of the elephant shark did not group with the zebrafish (**Figures 3B, C**).

To investigate whether mouse *MRAP2* undergoes alternative splicing similar to that reported in canine and human *MRAP2*, we analyzed annotated transcript variants in the mouse genome (GRCm39) (4, 49). As shown in **Supplementary Figure S1A**, three transcript isoforms of *Mrap2* were identified: *Mrap2-201* (ENSMUST00000049457.14), *Mrap2-202* (ENSMUST00000113149.8), and *Mrap2-203* (ENSMUST00000179313.3). All three isoforms share a conserved coding sequence (CDS) composed of four exons (ENSMUSE00000235341, ENSMUSE00000235357, ENSMUSE00000694502, and ENSMUSE00000694506), with divergence confined to their 5′ and 3′ untranslated regions (UTRs). Sequence alignment of the three isoforms revealed no variation in the open reading frame, suggesting that all three transcripts encode the same 207-amino acid MRAP2 protein (NP_001094952.2), as illustrated in **Supplementary Figures S1B–D**. Key functional motifs were conserved across all variants, including a predicted N-linked glycosylation site (Asn9), the LKAHKYS motif, a single transmembrane domain (TMD), and the YEYY motif. These features are hallmarks of MRAP2 proteins in other mammals, supporting structural conservation. In contrast to canine *MRAP2*, which produces two distinct isoforms through differential exon inclusion (e.g., *MRAP2a* from exons 2, 6, 7, 11 and *MRAP2b* from exons 3, 4, 6, 7, 11), no protein-coding alternative splicing was observed in mouse *MRAP2*. The murine isoforms lack exon-skipping events that would result in N-terminal or C-terminal differences. Therefore, the observed transcript variants represent UTR-level regulation rather than functional isoform diversity. These findings indicate that mouse *MRAP2* maintains a conserved protein structure across its transcript isoforms. It does not exhibit alternative splicing events generating different mouse *MRAP2* isoforms at the protein level, as seen in dogs or humans.

### Tissue distribution of *Mtnr1a*, *Mtnr1b* and *Mrap2* in mouse

**Figure 4:**
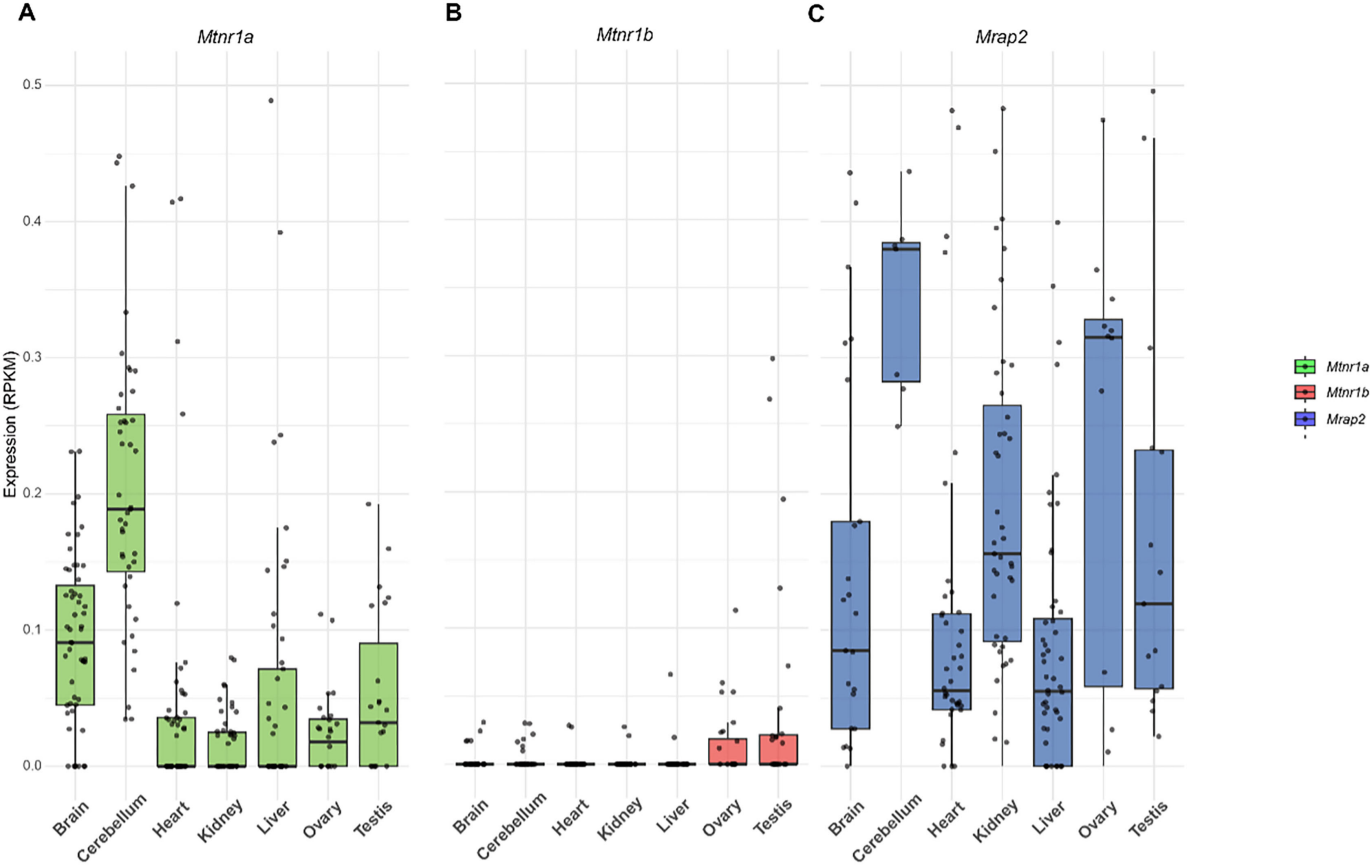
Tissue distribution of *Mtnr1a*, *Mtnr1b*, and *Mrap2* mRNA in mouse. **(A)** *Mtnr1a*, **(B)** *Mtnr1b*, and **(C)** *Mrap2* relative gene expression levels in different tissues. Each data point in the E-MTAB-6798 dataset represents a distinct biological sample. The number of replicates per tissue is as follows: brain (n = 11), cerebellum (n = 44), heart (n = 55), kidney (n = 51), liver (n = 58), ovary (n = 28), testis (n = 27), hindbrain (n = 43). A black line inside the box indicates the median, while the box edges denote the interquartile range (IQR); the whiskers extend to 1.5×IQR. Any data points outside this range are displayed individually.

Bulk RNA sequencing analysis of multiple mouse tissues revealed that melatonin receptors are expressed across various organs, including the cerebellum, brain, testis, kidney, ovary, heart, and liver (**Figures 4A, B**). Notably, Mtnr1a displayed the strongest expression in the brain and cerebellum, implying a key function in the central nervous system while suggesting activity in peripheral tissues (50). Notably, *Mtnr1a* showed overall higher expression compared to *Mtnr1b*. In addition, *Mrap2* was widely expressed across multiple organs (**Figure 4C**), consistent with previous reports highlighting its broad regulatory role in GPCR signaling (18). Interestingly, the expression patterns of *Mtnr1a*, *Mtnr1b*, and *Mrap2* were consistent in specific tissues, suggesting the possibility of functioning as a protein complex.

### MRAP2 interacts with MTNR1A and MTNR1B

**Figure 5:**
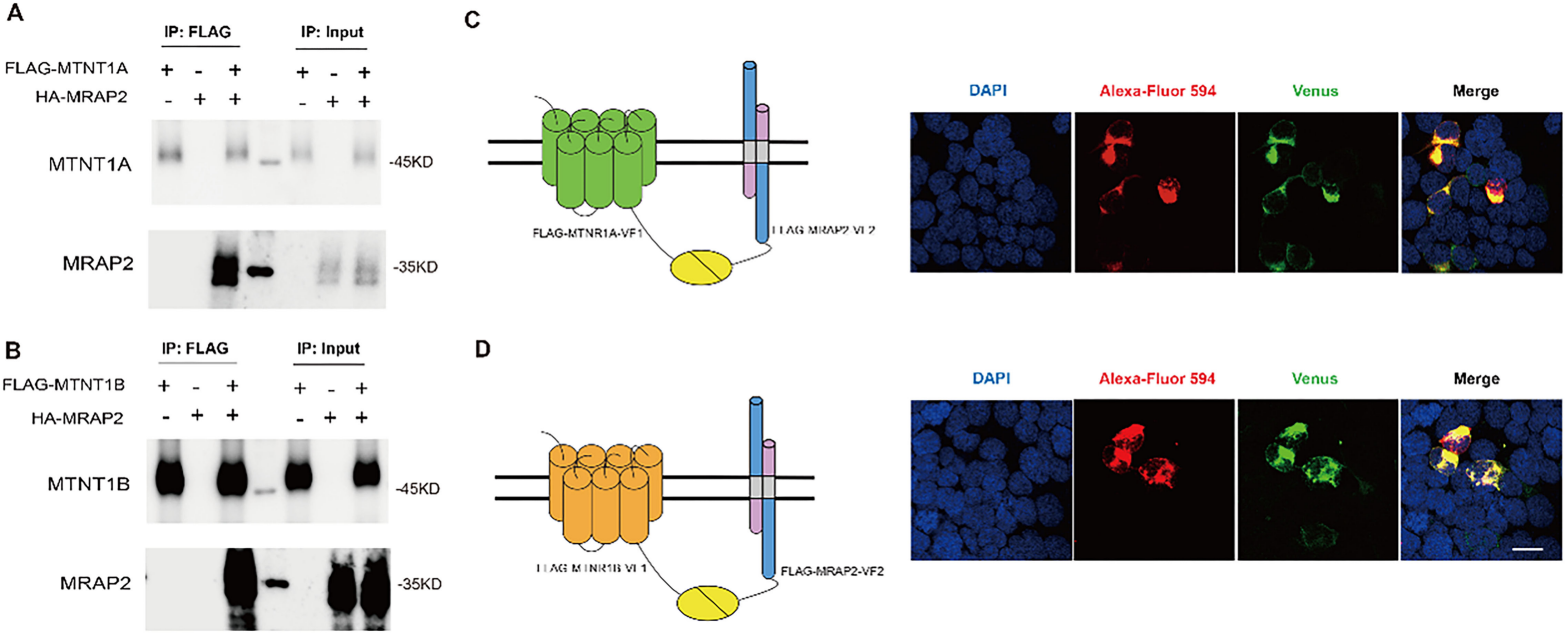
Interaction and co-localization of mouse MTNR1A/MTNR1B and MRAP2 *in vitro*. **(A)** Co-immunoprecipitation of 2xFlag‐MTNR1A with 3xHA‐MRAP2 in HEK293T cells. **(B)** Co-immunoprecipitation of 2xFlag‐MTNR1B with 3xHA‐MRAP2 in HEK293T cells. **(C)** Confocal microscopy of the co-localization of MTNR1A and MRAP2 on the plasma membrane. **(D)** Confocal microscopy of the colocalization of MTNR1B and MRAP2 on the plasma membrane. Nuclei stained with DAPI are shown in blue and Venus fluorescence in green. Surface expression of MTNR1A/MTNR1B, and MRAP2 is shown in red, detected by anti‐Flag antibody and secondary anti‐rabbit Alexa Fluro 594 (Abcam). Scale bar = 50μm.

The co-localization of MTNR1A and MRAP2, as well as MTNR1B and MRAP2, was first verified by bulk RNA-seq analysis. MRAP2, an accessory protein, could broadly regulate GPCRs that signal through Gs and Gq pathways, including melanocortin receptors (18, 51). This study explored MRAP2’s potential role in regulating melatonin receptors function linked to the Gi signaling pathway. We performed co-transfection with HA-MRAP2 and FLAG-MTNR1A or FLAG-MTNR1B plasmids in HEK293 cells to conduct the Co-immunoprecipitation (Co-IP) assay. FLAG-MTNR1A immunoprecipitated with HA-MRAP2 (**Figure 5A**). Similarly, a direct interaction between MTNR1B and MRAP2 was also observed (**Figure 5B**). Our results confirmed the protein interactions between both melatonin receptors and MRAP2.

We further utilized the BiFC to validate the interaction between melatonin receptors and MRAP2. When two proteins interact, their respective complementary Venus fragments bind together, forming a complete Venus protein that can be observed under a confocal microscope. An unmistakable Venus signal of MTNR1A and MRAP2 was shown on the cell membrane (**Figure 5C**). The phenomenon between MTNR1B and MRAP2 was similar to that of MTNR1A (**Figure 5D**), corroborating the Co-IP results.

### Pharmacological modulation of MTNR1A and MTNR1B by MRAP2

**Figure 6:**
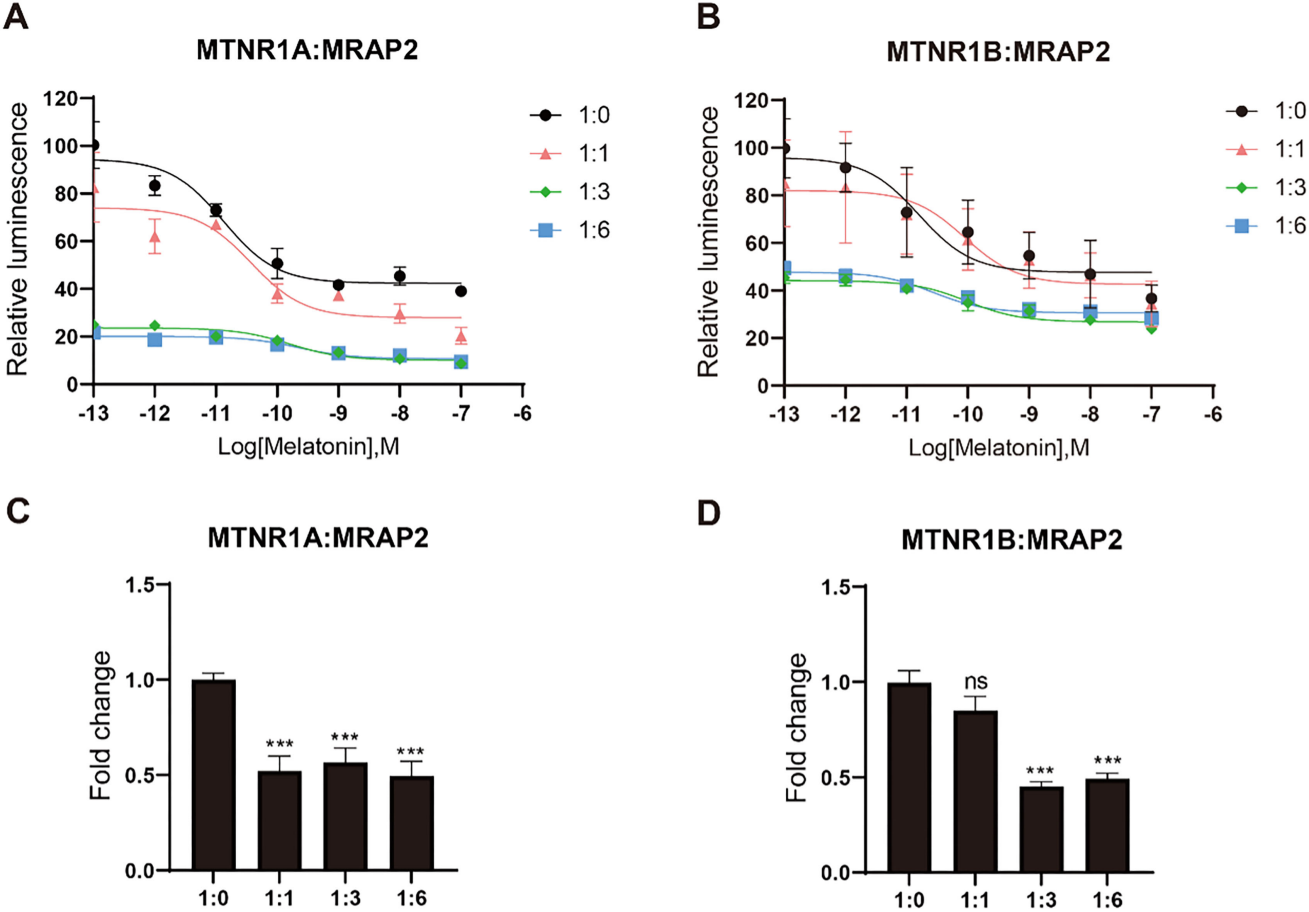
Pharmacological modulation of mouse MTNR1A and MTNR1B by melatonin. **(A)** and **(B)** show the effects of different stoichiometries of MRAP2 on the cAMP responses of MTNR1A or MTNR1B at different concentrations of melatonin (10^-13^ – 10^-7^ M), using the GloSensor cAMP assay. **(C)** and **(D)** show the constitutive activity affected by different stoichiometries of MRAP2 without ligand, using the GloSensor cAMP assay. Each bar represents the mean ± SEM of three biological replicates (n = 3). Constitutive activity statistical significance was evaluated between the baseline group (1:0) and each receptor stoichiometry group. One-way ANOVA was performed (***p < 0.001, ns, not significant).

After confirming the interaction between MRAP2 and both MTNR1A and MTNR1B, we further investigate whether MRAP2 modulates the pharmacological response of melatonin receptors upon ligand activation by the GloSensor detection assay. As melatonin concentration increased, it activated the downstream Gi signaling pathway of the melatonin receptors, resulting in diminished receptor quantities and reduced intracellular cAMP levels. For MTNR1A and MTNR1B, significant inhibitory effects were observed in the pharmacological curves upon transfection with 3-fold and 6-fold amounts of MRAP2 (**Figures 6A, B**). However, the logEC50 of the MTNR1A curve progressively increased with higher MRAP2 transfection levels, while no noticeable change in logEC50 was observed for MTNR1B (**Table 1**).

**Table 1:** Statistical analysis of ligand binding curves for mouse melatonin receptors with different dosage of MRAP2.

Analysis	MTNR1A:MRAP2	MTNR1B:MRAP2
LogEC50 1:0	-10.88 ± 0.35	-10.8 ± 0.91
LogEC50 1:1	-10.43 ± 0.53	-10.05 ± 1.11
LogEC50 1:3	-9.79 ± 0.46	-10.01 ± 0.88
LogEC50 1:6	-9.66 ± 0.71	-10.57 ± 0.88
*P*-value 1:0 vs 1:1	ns	ns
*P*-value 1:0 vs 1:3	***	**
*P*-value 1:0 vs 1:6	****	**

One-way ANOVA was applied in the statistical analysis. *, P < 0.05; **, P < 0.01; ***, P < 0.001; ****, P < 0.0001; ns, not significant.

In the absence of melatonin, transfection of MRAP2 at equimolar amounts with MTNR1A significantly reduced the constitutive activity of MRAP2, with no further decrease observed upon transfecting higher amounts of MRAP2 (**Figure 6C**). For MTNR1B, the constitutive activity decreased significantly as the expression level of MRAP2 increased at ratios of 1:3 and 1:6 (**Figure 6D**). Overall, MRAP2 exerted an apparent inhibitory effect on the constitutive activity of melatonin receptors and decreasing agonist-induced responses (**Figure 6**). This effect was likely due to the formation of a complex that altered the receptor conformation or increased its affinity for the ligand.

### MRAP2 regulates cell surface expression of MTNR1A and MTNR1B

**Figure 7:**
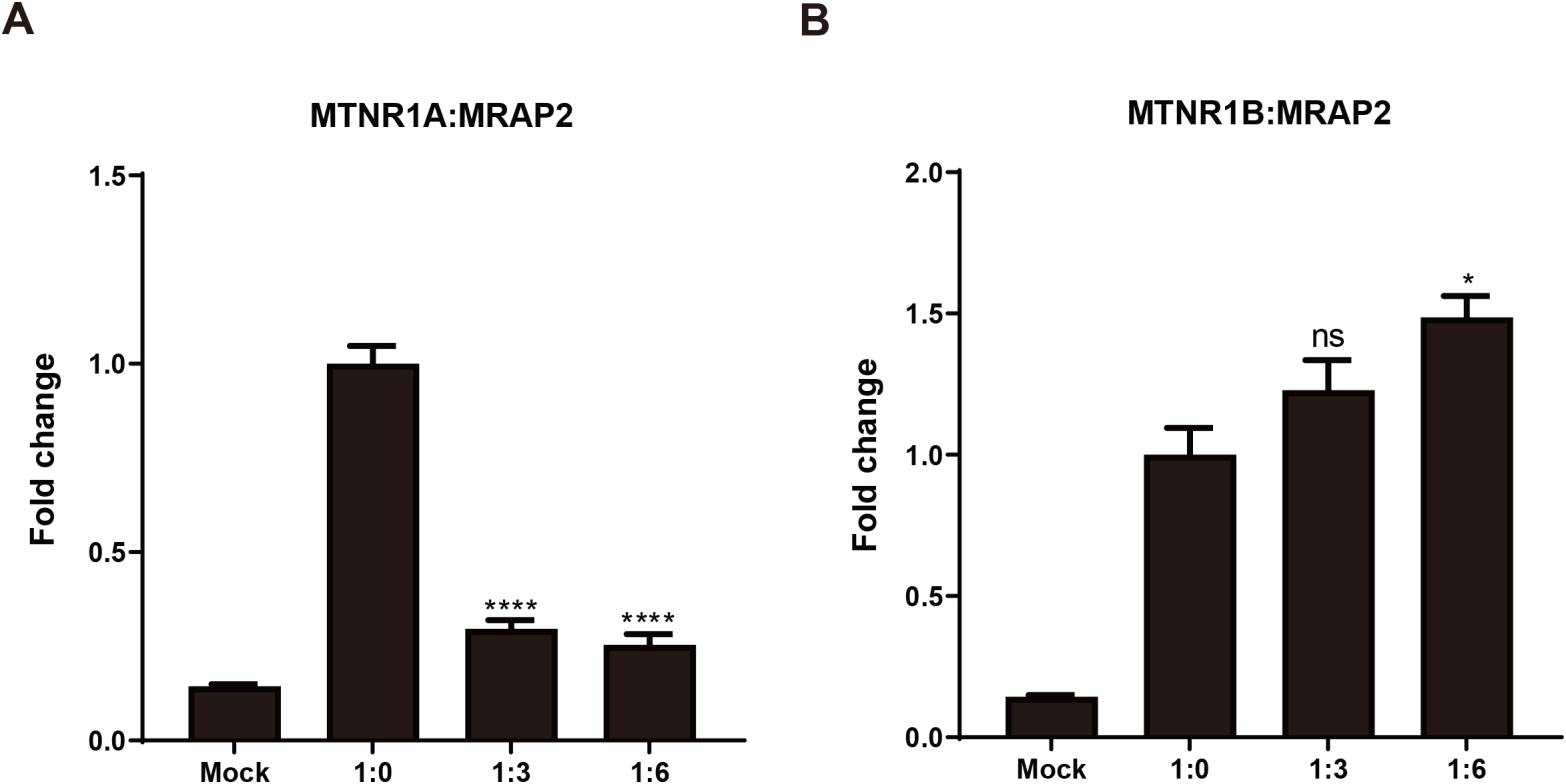
Modulation of the cell surface expression of mouse MTNR1A and MTNR1B by MRAP2. **(A)** Cell surface expression of mouse MTNR1A in presence of different amounts of MRAP2 (1:0, 1:3, 1:6). **(B)** Cell surface expression of mouse MTNR1B in presence of different amounts of MRAP2 (1:0, 1:3, 1:6). The cell surface expression of each receptor was presented as fold change of the measured surface epitopes level when the receptor was expressed alone. Each data represents mean ± SEM of three replicates (n = 3). One-way ANOVA with Tukey post-test was performed for all groups of data (*p < 0.05, ****p < 0.0001, ns, not significant).

To assess how mouse MRAP2 influences MTNR1A and MTNR1B localization at the plasma membrane, ELISA-based surface epitope detection was performed in HEK293T cells. This interaction implies that MRAP2 might control the migration of melatonin receptors to the cellular membrane. **Figure 7A** demonstrates a notable decrease in the cell surface expression of MTNR1A in the presence of MRAP2, indicating an inhibition of MTNR1A migration to the cellular membrane by MRAP2. In contrast, MTNR1B’s cell surface expression increased when co-expressed with MRAP2 at a 1:6 ratio, with no significant change at a 1:3 ratio (**Figure 7B**). Moreover, MRAP2’s presence enhanced MTNR1B’s cell surface expression in a dose-dependent manner. These distinct impacts of MRAP2 on melatonin receptor cell surface expression propose a multifaceted mechanism where MRAP2 serves as an accessory protein in regulating GPCR trafficking and post-translational translocation to the cell surface.

## Discussion

The circadian rhythm is known to be regulated by melatonin receptor activity (52, 53). The MTNR1A receptor inhibits neuronal activity, which is vital in regulating circadian rhythms, emotional regulation, cognitive function, and immune responses (54). The MTNR1B receptor participates in the secretion of neurotransmitters and hormones (55). Melatonin receptors, distributed across various tissues (**Figure 4**), synchronize central and peripheral oscillators, having been focused on neurodegenerative diseases and antidepressant therapies (50). Meanwhile, the broader tissue distribution of MTNR1B (e.g., liver, kidney) supports its participation in peripheral functions such as insulin sensitivity and hormone release (56). The ubiquitous expression of MRAP2 corresponds to its broad role as a universal GPCR modulator (17, 57). Co-expression of MTNR1A/B and MRAP2 in overlapping tissues (e.g., brain) suggests cooperative synchronization, potentially forming receptor-MRAP2 complexes to modulate signaling cascades (**Figure 4**). Despite classifying melatonin receptors into three types, the MT3 subtype has been recognized as quinone reductase 2 (NQO2) rather than a GPCR, showing a relatively low affinity for melatonin (58). Therefore, MT3 is not the focus of this study.

Synteny and phylogenetic analyses revealed the evolution of *MTNR1A, MTNR1B*, and *MRAP2* across vertebrates, including mammals, birds, amphibians, and teleosts. The conserved synteny observed for *MTNR1A, MTNR1B, and MRAP2* among vertebrates, excluding teleosts, underscores their evolutionary stability, particularly prominent in mammals (**Figure 2**). MTNR1A shows significant sequence conservation, while MTNR1B and MRAP2 display increased variability, especially at their N- and C-termini (**Figures 1A–C**). This divergence is associated with the broader regulatory roles of MRAP2 in GPCR signaling. The variability in adjacent genes, particularly in zebrafish, may indicate specific adaptations to environmental or physiological factors unique to fish species teleosts (59). Teleosts exhibit a robust background adaptation mechanism, with evidence that the pineal gland contributes to pigmentation regulation in response to environmental cues via a pineal-hypothalamus neuroendocrine axis (60). The variability in the adjacent genes, particularly in zebrafish, may also reflect specific adaptations to environmental or physiological factors unique to teleosts. Nonetheless, the transmembrane core and intracellular signaling mechanisms of GPCRs have remained conserved (59), as confirmed by multiple protein sequences alignment for MTNR1A and MTNR1B (**Figures 1A, B**). Furthermore, the multiple sequence alignment for MTNR1A, MTNR1B, and MRAP2 reveals more pronounced amino acid distinctions starting from the clawed frog (**Figure 1**). Multiple paralogs of MTNR1A and MTNR1B in zebrafish suggest potential functional diversification within the species, indicative of teleost-specific adaptations possibly associated with environmental pressures or metabolic demands. In contrast to teleosts, cartilaginous fish like elephant sharks lack MTNR1A/B paralogs, suggesting that melatonin receptor diversification coincided with vertebrate territorialization. In the more primitive species of sea lamprey, it has been demonstrated that slMRAP2 exerts distinct pharmacological regulation on the earliest melanocortin receptors, slMCa and slMCb (61).

MRAP2, a crucial regulator of energy balance, modulates diverse GPCRs in the central nervous system (16). MRAP2 alters biased signaling of GHSR1a, suppresses PKR1 trafficking and signal transduction, and modulate OX1R function (62). MTNR1A, MTNR1B, and MRAP2 are also co-expressed in the brain region (**Figure 4**). We confirmed the interaction between MRAP2 and MTNR1A and MTNR1B using co-immunoprecipitation (Co-IP) assays. The immunoprecipitation demonstrated a direct physical association between MRAP2 and the melatonin receptors (**Figures 5A, B**). This interaction was further supported by the BiFC assay, which visualized the combination between melatonin receptors and MRAP2 at the cell membrane (**Figures 5C, D**). This study further elucidates the functional impact of MRAP2 modulation of melatonin receptor signaling. MRAP2 inhibited both constitutive activity and ligand-induced intracellular cAMP levels (**Figure 6**), suggesting that MRAP2 may influence melatonin receptor signaling efficiency, potentially through changes in receptor trafficking or membrane localization, rather than direct modulation of ligand binding or constitutive activity.

The obvious co-localization of these proteins at the plasma membrane suggests that MRAP2 is directly involved in the trafficking and membrane localization of melatonin receptor signaling. Our results find that MRAP2 can bidirectionally regulate GPCR trafficking. MRAP2 hindered the movement of MTNR1A to the cell membrane while promoting the surface display of MTNR1B in a manner dependent on the dosage (**Figure 7**). Interestingly, the receptors in our experiments were not stimulated with agonists, ruling out ligand-induced receptor internalization or desensitization as drivers of surface expression changes. So, the changes in surface levels are not likely caused by internal movement or loss of receptors after activation. These changes are more likely caused by how MRAP2 affects the way receptors move out from inside the cell (63). When forward movement (anterograde trafficking) is blocked, GPCRs may not reach the surface, which can happen if the receptors are folded incorrectly, held inside the endoplasmic reticulum (ER), or do not pass through the Golgi (64). Depending on the type, MRAP2 might change one or more of these steps to help or block the movement of receptors. Desensitization means the receptors react less after being exposed to a ligand for a long time. Nevertheless, this process usually happens only after a ligand activates the receptor, which did not happen in our tests (54, 65).

These results align with emerging data indicating that MRAP2 can regulate GPCRs bidirectionally. MRAP2 inhibits the constitutive activity of MC4R and enhances the cell surface expression of MC2R (2). It influences the pharmacological function of MC5R in a way that varies by species - impeding trafficking in humans and promoting trafficking in teleosts, possibly due to distinct interactions between the receptor and MRAP2 variants (14). Notably, the rise in MTNR1B surface levels contrasts with MRAP2-induced reduction of MC4R and MC5R in mammalian models, implying diverse regulatory pathways may have evolved for melatonin receptors. The conserved YEYY and LKAHKYS motifs in MRAP2 (**Supplementary Figures S1B–D**) likely mediate these effects by altering receptor-ligand affinity or coupling efficiency with G proteins (2). These conserved patterns suggest MRAPs may serve as universal modulators of GPCR homeostasis, though their molecular mechanisms likely diverge.

The bidirectional regulation of constitutive activity by MRAP2 parallels findings in other GPCR systems. Co-expression of MRAP2 with CRHR1 reduces basal cAMP production, mirroring our observation of suppressed constitutive activity in MTNR1A (18). Conversely, MRAP2 increases MC5R maximal binding capacity without altering ligand sensitivity in ricefield eels, a phenomenon analogous to its enhancement of MTNR1B signaling efficacy (66). This dual modulation—reducing basal cAMP levels while amplifying ligand-dependent signaling—parallels its effects on CRHR1 and MC5R (67, 68). Structural studies reveal that MRAP1 stabilizes MC2R in an active conformation through N-terminal interactions, while MRAP2 disrupts MC5R homodimerization to alter signaling (66). Whether similar structural rearrangements underlie MRAP2’s effects on melatonin receptors warrants further investigation.

Several drugs targeting melatonin receptors have been developed, primarily consisting of endogenous melatonin agonists and receptor antagonists, including ramelteon, tasimelteon, suvorexant, and rozerem (69, 70). Existing drugs targeting melatonin receptors are known to cause side effects, including headaches and gastrointestinal discomfort. In our study, MRAP2 is a crucial accessory protein, influencing receptor activity, membrane localization, and ligand responsiveness of melatonin receptors MTNR1A and MTNR1B. However, future studies should investigate the *in vivo* effects of MRAP2 modulation on melatonin receptor activity. Our study demonstrates that MRAP2 functionally regulates the melatonin receptor family, expanding the range of GPCRs it can modulate and reaffirming its crucial role in energy metabolism. These results enhance our comprehension of the molecular pathways governing melatonin receptor physiology and present novel opportunities for creating substitute therapies that could alleviate the adverse effects of existing treatments.

## Data Availability

Publicly available datasets were analyzed in this study. This data can be found here: RNA-seq data (accession code: E-MTAB-6798) from the ArrayExpress database.

## Supplementary material

Supplementary Figure 1 Alternative splicing analysis of murine MRAP2 transcripts. **(A)** Exon–intron organization of three Mrap2 transcript variants in Mus musculus (Mrap2-201, Mrap2-202, and Mrap2-203) as annotated in Ensembl GRCm39. Protein-coding exons (dark yellow) and UTRs (light yellow) are shown for each isoform, with connecting lines representing splicing junctions. All three transcripts share the same coding sequence composed of four exons, while transcript diversity arises from alternative 5′ and 3′ UTR regions. **(B–D)** cDNA sequences of murine MRAP2 transcripts, Mrap2-201**(B)**, Mrap2-202**(C)**, and Mrap2-203**(D)** with coding sequence (CDS) and conserved motifs annotated. Colored boxes highlight key domains: N-linked glycosylation site (Black), YEYY motif (red), LKAHKYS motif (blue), and transmembrane domain (green). Asterisk (*) shows the stop codon.

Supplementary Table 1 All species and sequence information utilized for sequence alignment, phylogenetic tree and synteny analysis.
